# Handling incidental findings in neuroimaging research in Japan: current state of research facilities and attitudes of investigators and the general population

**DOI:** 10.1186/1478-4505-12-58

**Published:** 2014-10-06

**Authors:** Misao Fujita, Yoshinori Hayashi, Shimon Tashiro, Kyoko Takashima, Eisuke Nakazawa, Akira Akabayashi

**Affiliations:** Uehiro Research Division for iPS Cell Ethics, Center for iPS Cell Research and Application (CiRA), Kyoto University, The Kyoto Technoscience Center #3, 14, Yoshidakawara-cho, Sakyo-ku, Kyoto, 606-8305 Japan; College of Letters, Human Studies Program, Ritsumeikan University, 56-1 Toji-in Kitamachi, Kita-ku, Kyoto, 603-8577 Japan; Office for Promoting Medical Research, Showa University, 1-5-8 Hatanodai, Shinagawa-ku, Tokyo, 142-8555 Japan; Department of Biomedical Ethics, The University of Tokyo Graduate School of Medicine, 7-3-1 Hongo, Bunkyo-ku, Tokyo, 113-0033 Japan

**Keywords:** General population, Incidental findings, Investigators, Japan, Neuroimaging, Questionnaires

## Abstract

**Background:**

To establish appropriate measures that deal with incidental findings (IFs), the neuroscience community needs to address various ethical issues. The current state of research facilities regarding IFs and investigator attitudes as well as potentially eligible research participants must be assessed prior to future discussions and before the development of policies and guidelines. To this end, we conducted two questionnaire surveys to clarify i) how IFs are addressed at neuroimaging research facilities in Japan and ii) the views of investigators and potential research participants regarding the handling of IFs.

**Methods:**

Thirty-one principal investigators (PIs) involved in the Strategic Research Program for Brain Sciences (SRPBS), a government-funded project, were asked to fill out a questionnaire regarding ways IFs were handled at the facility. A total of 110 investigators engaged in SRPBS tasks, including 31 PIs who participated in the research facility survey and researchers conducting studies under the management of the PIs, and 500 individuals from the general public (i.e., general population) were asked to select the most appropriate way to deal with IFs in two scenarios, namely the medical school and humanities and social sciences department scenarios.

**Results:**

More than 40% of PIs responded that they did not know or were unsure of what type of approach was employed to handle IFs at their research facilities. Nevertheless, they were willing to improve the current status if sufficient resources were provided. With regard to specialist involvement, 37.7% of investigators responded that it was appropriate to have a specialist check all images in the medical school scenario, whereas 13.3% responded that such involvement was appropriate in the humanities and social sciences department scenario. In contrast, 76.1% and 61.0% of the general population indicated that specialist involvement was appropriate in the medical school and humanities and social sciences department scenarios, respectively. These results show that expectations of the general population exceed those of investigators regarding measures to address IFs. Both investigators and the general population demanded more responsibility from PIs at medical institutions, compared to PIs at non-medical institutions.

**Conclusions:**

Based on our preliminary results, we recommended that a licensed physician perform a screening test to appropriately examine clear abnormalities. These recommendations were implemented by the SRPBS as guidelines for handling IFs in national research projects in Japan.

## Background

An incidental finding (IF) is defined as “*a finding concerning an individual research participant that has potential health or reproductive importance and is discovered in the course of conducting research but is beyond the aims of the study*” [1]. In neuroimaging research, IFs include brain tumors, cerebral aneurysms, or asymptomatic vascular lesions, which are not directly related to research aims but are found by magnetic resonance imaging (MRI) or research MRI sequences. One study reported that the frequency of brain-related IFs that are found by MRI or functional magnetic resonance imaging (fMRI) studies is 13% to 84% [1]. In that study, 1.2% of findings required immediate referral for clinical evaluation, whereas 13% to 40.4% of findings did not [1]. Recently, the neuroscience research community has aimed to establish measures to handle such findings, as various ethical questions regarding IFs have been raised [2].

Research MRI images typically have lower resolution and contrast [3], and are often unsuitable for clinical diagnoses. Moreover, opportunities for non-physician investigators who are untrained in image evaluation to conduct neuroimaging research in non-medical areas (e.g., cognitive psychology and behavioral economics) have increased. This can lead to an increased risk of false-negative errors (i.e., a disease is overlooked) and false-positive errors (i.e., a disease is inaccurately diagnosed). False-negative errors can lead a participant to ignore the early symptoms of a disease and thereby aggravate his or her condition. In fact, the majority of healthy adults who participate in neuroimaging studies expect the study to discover any abnormality that exists [4]. In contrast, false-positive errors might significantly burden participants (i.e., mentally, economically, and temporally) until the absence of disease is confirmed. Indeed, one study revealed that subsequent examination confirmed all detected IFs to be false positives [5].

There is a general consensus within the research community that IFs should be addressed appropriately [6, 7]. However, there exists evidence that IFs are handled differently across research facilities. According to a survey conducted by Lawrenz and Sobotka, informed consent forms that mentioned IFs were used in approximately 9%, 25%, 11%, and 37% of cases at federal institutions, relevant conferences, universities, and other facilities, respectively, and the contents differed for each form [8]. An Internet survey conducted by Illes et al. showed that more than 80% of investigators encountered IFs during their studies; however, only half of these investigators indicated that a standard procedure existed for addressing IFs at their research facilities [9]. With respect to specialist (neuroradiologist) involvement, their responses ranged from “checks all images” and “checks only equivocal findings” to “checks no images at all” [9].

Opinions regarding appropriate measures to be taken by principal investigators (PIs) are also divided. Royal and Peterson are against specialist involvement, as they believe such involvement may increase the risk of false-positive errors. In other words, a specialist, who is concerned “*about medicolegal liability from failing to identify a serious IF*,” may “*err on the ‘safe side’ by recommending further consultation*” [10]. According to Richardson, actively searching for abnormal findings violates participant privacy, although the PI is responsible for providing “ancillary care” for a disease discovered during a study [11]. Another view suggests that the reading of all images or use of clinical scans by specialists is not always required [2, 12]. In contrast, Milstein asserts that the PI should provide the highest level of care (e.g., detailed examinations and the use of clinical devices), because otherwise, s/he may be sued if a disease worsens or the participant develops a disorder during the course of research [13].

Given that various approaches are adopted by research facilities with differing opinions among scholars, the recommendations summarized during the Detection and Disclosure of Incidental Findings in Neuroimaging Research workshop, which was held in 2005 by the National Institutes of Health (NIH) and Stanford University, represented a significant breakthrough [14]. Five options were provided for addressing IFs so that investigators can implement the most appropriate option for them. In 2008, Wolf et al. provided more detailed recommendations regarding the ethical and legal aspects of IFs in neuroimaging and genetic/genomic research [1]. Furthermore, in October 2012, the NIH and a working group supported by US and Canadian government agencies discussed measures to address IFs in neuroimaging research, and published the results online [15, 16]. However, directly adopting these recommendations in countries other than the US and Canada would require careful consideration.

In Japan, the Ministry of Education, Culture, Sports, Science, and Technology (MEXT) launched the Strategic Research Program for Brain Sciences (SRPBS) in 2008 to strategically advance neuroscience research [17]. Government research conducted with public funds includes many neuroimaging studies that use MRI and fMRI techniques. However, the only guidance available regarding IFs in neuroimaging research is found in the Guidelines on Ethical Issues of Noninvasive Research on Human Brain Function, which was revised in 2009 by the Japanese Neuroscience Society [18]. These guidelines deem it appropriate to consult a specialist prior to disclosing an IF to a participant, but the reasons for this approach are not mentioned. Moreover, these guidelines only have binding power on association members. In general, there has been little discussion regarding how IFs should be addressed in Japan [19, 20].

To facilitate future discussions and develop additional policies or guidelines, we must first assess the current state of relevant research facilities, and clarify attitudes of investigators and potentially eligible research participants regarding IFs. However, few empirical studies have investigated the actual procedures for addressing IFs at research facilities in Japan [19, 20]. Moreover, the focus has been placed on conducting surveys of investigators in most international studies [9, 21], or of participants who have already taken part in research [4, 22, 23], and few studies have investigated the attitudes of potential research participants regarding IFs procedures. Thus, whether their attitudes differ from those of investigators is unclear. Therefore, the present study used two questionnaires to survey the following: i) how IFs are addressed at neuroimaging research facilities in Japan, and ii) the views of investigators and potential research participants regarding the handling of IFs. As issues surrounding IFs are still somewhat unexplored in Japan, we used a modified version of the five options established by Illes et al. [14], along with two scenarios, to help participants better understand the questionnaires.

## Methods

### Research participants

#### Research facility survey

PIs of 31 research facilities involved in the SRPBS between 2008 and 2010 were recruited for this survey. These research facilities were engaged in one of the following tasks: “Development of brain-machine interfaces,” “Development of highly original animal models,” and “Development of technologies to measure and assist brain mechanisms that support social behaviors.” The questionnaires were mailed according to the address of each research facility and the names of corresponding PIs listed on the SRPBS website [17].

#### Investigators/general population survey

Study participants were a total of 110 investigators engaged in SRPBS tasks, including 31 PIs who participated in the research facility survey and researchers conducting studies under the management of the PIs. A list of their addresses and names was obtained, and the questionnaires mailed, upon approval of the SRPBS. The general population in this survey refers to registered members of the Japan Management Association Research Institute, which recruits volunteers who participate in various studies [24]. A questionnaire was uploaded onto the institute’s website, and registered volunteers were able to choose whether or not to answer the questionnaire. The questionnaire remained available until 500 responses were received. We collected 100 from each age group ranging from 20 to 60 years (250 from each gender) to avoid sampling biases.

### Questionnaires

Between October and November 2010, the PIs, investigators, and the general population were asked to complete an anonymous self-administered questionnaire. IFs were defined as follows:

“*‘Incidental findings (IFs)’ refer to unexpected health problems (e.g., brain tumors, cerebral aneurysms, or asymptomatic vascular disorders) discovered by coincidence during a study on a research participant’s MRI and/or fMRI brain images. The frequency of IFs has been reported to be 13–84%, and 1.2% of IFs require immediate examination. Study participants benefit from these IFs because they are made aware of their disease.*

*However, imaging devices used for research purposes may result in false-negative (i.e., judged healthy despite having a disease) or false-positive (i.e., judged to have a disease despite being healthy) errors. In false-positive cases, participants may experience unnecessary stress and economic burden until the absence of disease is confirmed.*”

The survey respondents were also informed that MRI is an examination method that uses a computer to render images of cross-sections of the body and that fMRI visualizes brain activity using MRI. It took about 5 to 10 min to complete the questionnaires. Only the general population received a reward in the form of points (approximately 20 to 30 points, which are exchangeable for money at a rate of 1 yen/point) from the research firm. This system is widely implemented by research firms in Japan.

#### Research facility survey

In the questionnaire, we asked PIs i) questions regarding basic characteristics, ii) how IFs are addressed at the research facility (“At the research institution to which you belong, which of options 1–6 (Table 1) is currently employed as a means of addressing IFs?”), and iii) what would be the appropriate way to address IFs if sufficient resources were available (“If the research institution to which you belong had adequate resources (e.g., personnel, equipment, and research funds) to address IFs, which of options 1–6 do you think would be most appropriate in terms of the extent of the response?”). These six options were obtained by reorganizing the five options established by Illes et al. [14] to facilitate statistical analyses.

**Table 1:** **Six options for addressing incidental findings (IFs)**

Option 1	When obtaining informed consent (IC), the possibility of IFs is not explained at all to the participant.
Option 2	When obtaining IC, the participant is informed that “IFs may be discovered, but will not be explained even if they are found.”
Option 3	When obtaining IC, the participant is informed that “IFs may be discovered, and if the principal investigator (PI) considers them suspect, s/he will notify the participant. However, a specialist (radiologist) does not check the images.”
Option 4	When obtaining IC, the participant is informed that “if IFs are suspected, a specialist (radiologist) will be asked to check the images. If a genuine problem appears to exist, the PI will notify the participant.” The cost for specialist consultation is obtained from the PI’s research funds.
Option 5	When obtaining IC, the participant is informed that “a specialist (radiologist) will check all images to discover IFs. If a genuine problem appears to exist, the PI will notify the participant.” The cost for specialist consultation is obtained from the PI’s research funds.
Option 6	When obtaining IC, the participant is informed that “a clinical device with more precision than that used for research will be initially used to detect IFs. A specialist (radiologist) will check all images, and the PI will notify the participant when a firm diagnosis is established.” The costs for the device and specialist consultation are obtained from the PI’s research funds.

#### Investigators/general population survey

The questionnaire included i) basic characteristic questions, ii) a scenario of an abundantly funded neuroimaging research study of patients at a medical school, which was conducted by a PI with a physician’s license (Medical school scenario), and iii) a scenario of a poorly funded neuroimaging research study of healthy individuals in a humanities or social sciences department, which was conducted by a PI without a physician’s license (Humanities and social sciences department scenario). We employed these two scenarios based on a previous study showing that attitudes of research participants toward IFs vary depending on whether the research context is medical or non-medical [4]. For ii) and iii), we asked all respondents to select the most appropriate way to address IFs from the six options. In addition, following Kirschen et al. [4], we asked the general population about their iv) expectations regarding image assessment in neuroimaging research.

#### Medical school scenario

A PI who is employed at a *medical school-affiliated university hospital* is planning a study to clarify the relationship between a person’s emotions and neural activity. Fifty *patients commuting to the affiliated hospital* (who are at least 20 years old) are recruited as participants. To measure neural activity, the plan is to use an fMRI device that has a lower precision than does a device that would be used in the clinical context. The PI *is a licensed physician* who has previously conducted numerous studies using fMRI devices. The allowable expenditure for this study is *10 million yen per year*. What approach should the PI adopt to address IFs?

#### Humanities and social sciences department scenario

A PI who is employed at a *humanities and social sciences department* of a university is planning a study to clarify the relationship between a person’s emotions and neural activity. Fifty *healthy students from the university* (who are at least 20 years old) are recruited as participants. To measure neural activity, the plan is to use an fMRI device that has a lower precision than does a device that would be used in the clinical context. Although *not a licensed physician*, the PI has previously conducted numerous studies using fMRI devices. The allowable expenditure for this study is *1 million yen per year*. What approach should the PI adopt to address IFs?

### Statistical analyses

We performed Wilcoxon rank sum and signed rank sum tests using SAS version 9.1. To test for significance, we used a two-tailed test with a criterion level of 5%.

### Ethical considerations

This study was approved by the Ethics Committee of the Tokyo University Graduate School of Medicine and Faculty of Medicine.

## Results

### Research facility survey

**Figure 1:**
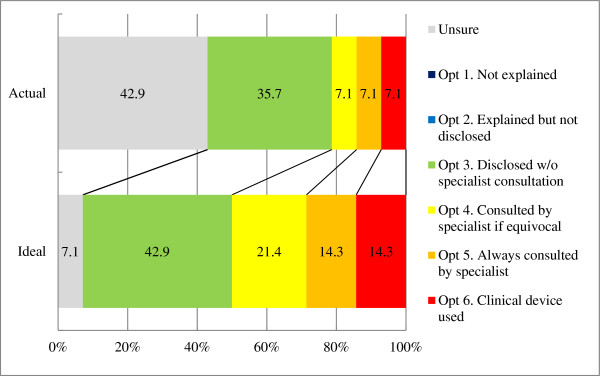
**Comparison of actual and ideal treatments of incidental findings.** Calculations were based on data obtained from the 14 principal investigators who conduct human research using imaging devices.

We mailed questionnaires to 31 PIs and obtained responses from 23 (response rate, 74.1%; Table 2). Of the 14 PIs who were conducting human research using imaging devices at their research facilities, 6 (42.9%) reported prior experience with IFs. These findings included brain tumors, cerebrovascular disorders, cerebral aneurysms, arachnoid cysts, and sinusitis. Figure 1 shows the current and ideal approaches for handling IFs according to the answers provided by the 14 PIs. Six (42.9%) answered that they were unsure of whether institutional guidelines exist, and 5 (35.7%) selected option 3 (Table 1) as the current institutional policy. However, half of the PIs indicated that specialist involvement would be appropriate (option 4 or higher) if resources were available. None of them currently adopted option 1 or option 2, or considered these options ideal. A comparison of responses concerning the current approach and those concerning the ideal approach using the Wilcoxon signed rank sum test revealed a significant difference (*P* =0.0156).

**Table 2:** **Basic characteristics**

		PIs n =23 (%)	Investigators n =69 ^b^(%)	General population n =500 (%)
Sex	Male	22 (95.7)	65 (94.2)	250 (50.0)
	Female	1 (4.4)	4 (5.8)	250 (50.0)
Age range (years)	20–29	0 (0)	0 (0)	100 (20.0)
	30–39	2 (8.7)	14 (20.3)	100 (20.0)
	40–49	10 (43.5)	25 (36.2)	100 (20.0)
	50–59	8 (34.8)	25 (36.2)	100 (20.0)
	>59	3 (13.0)	5 (7.3)	100 (20.0)
Affiliation	University medical school	10 (43.5)	31 (45.6)	
	University science and engineering department	2 (8.7)	10 (14.7)	
	University humanities and social sciences department	1 (4.4)	2 (2.9)	
	Non-university research institution	4 (17.4)	13 (19.1)	
	Company	3 (13.0)	4 (5.9)	
	Other	3 (13.0)	8 (11.8)	
Licensed physician	Yes	10 (43.5)	35 (50.7)	
	No	13 (56.5)	34 (49.3)	
Use of neuroimaging devices	Yes	16 (69.6)	26 (37.7)	
	No	7 (30.4)	43 (62.3)	
Research subjects	Non-human	8 (34.8)	34 (50.0)^c^	
	Human	15 (65.2)	34 (50.0)^c^	
	w/ patients	12 (80.0)	25 (75.8)^d^	
	w/o patients	3 (20.0)	8 (24.2)^d^	
	w/ IF experience	6 (42.9)^a^	10 (30.3)^d^	
	w/o IF experience	8 (57.1)^a^	23 (69.7)^d^	
Guidelines on IFs available	Yes	4 (18.2)	8 (11.6)	
	No	12 (54.6)	27 (39.1)	
	Unsure	6 (27.3)	34 (49.3)	
Experience with tests that employ neuroimaging technology	Yes			145 (29.0)
	No			355 (71.0)
Experience participating in studies that use neuroimaging technology	Yes			8 (1.6)
	w/ IF experience			2 (25.0)
	w/o IF experience			6 (75.0)
	No			492 (98.4)

^a^Of the 15 PIs who were conducting research on humans, one did not respond and was thus excluded from the calculations.

^b^Of the 70 investigators, one with incomplete information was excluded from the calculations.

^c^Of the 69 investigators with complete information, one without responses was excluded from the calculations.

^d^Of the 34 investigators who were conducting research on humans, one without responses was excluded from the calculations.

### Investigators/general population survey

Of the 110 investigators to whom questionnaires were mailed, 70 responded (response rate, 63.6%; Table 2). Of the 33 investigators who were conducting human research, 10 (30.3%) reported having experience with IFs. These findings included brain tumors, normal pressure hydrocephalus, lacunar infarctions, venous malformations, cavernous hemangiomas, cerebral aneurysms, arachnoid cysts, carotid artery malformations, and sinusitis. Of the 500 individuals from the general population who answered the web-based questionnaire, 8 (1.6%) had prior experience participating in research using neuroimaging technology. Of these, 2 were found to have central nervous system vasculitis and cerebral infarction during the course of study participation.

#### Medical school scenario

**Figure 2:**
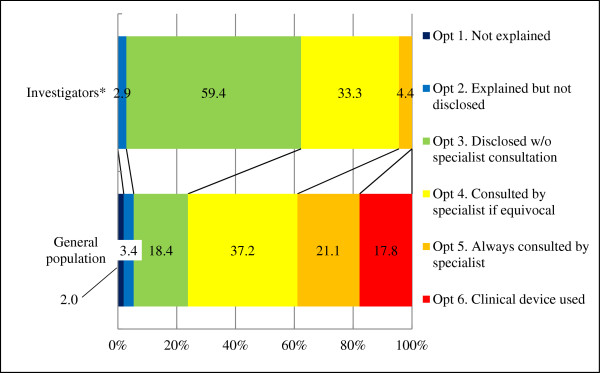
**Appropriate approaches that principal investigators should adopt according to the medical school scenario.** *Of the 70 investigators, one without complete information was excluded from the calculations.

More than 97% (67/69) of investigators considered it appropriate to notify the participant if IFs were discovered (option 3 or higher; Figure 2). However, 62.3% (43/69) of investigators considered it unnecessary to have a specialist check the images (option 3 or lower), whereas 37.7% indicated that having a specialist check the images was desirable (26/69; option 4 or higher). Only 4.4% (3/69) of investigators responded that it was desirable to have a specialist check all images (option 5 or higher), and none supported the use of more precise clinical devices for the purpose of detecting IFs (option 6). The Wilcoxon rank sum test revealed that the responses did not differ significantly according to whether an investigator was a licensed physician, was affiliated with a medical school, had used imaging equipment, had conducted human research, or had guidelines for addressing IFs.

In contrast, only 23.8% (119/500) of the general population considered it unnecessary to have a specialist check the images (option 3 or lower), whereas 76.1% (381/500) indicated that having a specialist check the images (option 4 or higher) was desirable. The proportion of individuals who responded that it was desirable to have a specialist check all images (option 5 or higher) was 38.9% (195/500), and that of those who supported the use of clinical devices (option 6) was 17.8% (89/500). A significant difference was detected (*P* <0.0001; Wilcoxon rank sum test) in responses between investigators and the general population.

#### Humanities and social sciences department scenario

**Figure 3:**
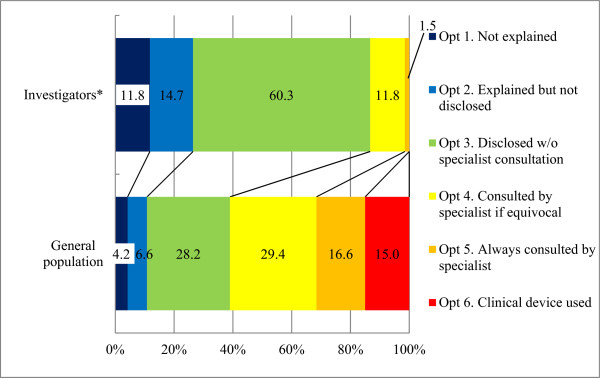
**Appropriate approaches that principal investigators should adopt according to the humanities and social sciences department scenario.** *Of the 70 investigators, two without complete information were excluded from the calculations.

Approximately 75% (50/68) of investigators considered it important to notify the participant if IFs were discovered (option 3 or higher; Figure 3). However, 86.8% (59/68) of investigators considered it unnecessary to consult a specialist (option 3 or lower). Only 13.3% (9/68) of investigators indicated that a specialist should check the images (option 4 or higher), and none supported the use of clinical devices (option 6). The Wilcoxon rank sum test revealed that responses did not differ significantly according to whether an investigator was a licensed physician, was affiliated with a medical school, had used imaging equipment, had conducted human research, or had guidelines for handling IFs.

Among the general population, 39.0% (195/500) considered specialist consultation unnecessary (option 3 or lower), whereas 61.0% (305/500) responded that it was desirable (option 4 or higher). Of these, approximately half (31.6%) indicated that a specialist should check all images (option 5 or higher), and 15% (75/500) supported the use of clinical devices (option 6). A significant difference was detected (*P* <0.0001; Wilcoxon rank sum test) in responses between investigators and the general population.

#### Comparison of the medical school and humanities and social sciences department scenarios

A significant difference (*P* <0.0001; Wilcoxon signed rank sum test) was observed when investigator responses were compared between the medical school and humanities and social sciences department scenarios. Similarly, a significant difference was observed in responses among the general population (*P* <0.0001).

#### General population’s view on handling IFs

In response to the question, “How do you think investigators verify whether a participant has an abnormality during a research study using MRI or fMRI,” 80.8% (404/500) of the general population indicated that they expected specialist involvement (Table 3).

**Table 3:** **Expectations of the general population toward image evaluation in neuroimaging research (n =500)**

Modes of evaluation	n (%)
No one checks the images	41 (8.2)
PI checks the images	55 (11.0)
Specialist checks the images only when there is a suspected finding	294 (58.8)
Specialist checks all images	90 (18.0)
Specialist uses a clinical device to check all images	20 (4.0)

## Discussion

This study is the first to assess the procedures for addressing IFs in neuroscience research facilities in Japan. Unlike previous studies [4, 9, 21–23], we compared the views of investigators and the general population regarding the handling of IFs. Moreover, the policy regarding IFs was implemented by the SRPBS, which took into consideration the preliminary results of this study.

### Current state of neuroimaging research facilities

Of the 14 research facilities that conduct neuroimaging research on humans, 40% had previously encountered IFs. Nonetheless, more than 40% of PIs indicated that they did not know or were unsure of what type of approach was employed at their facilities. This finding is consistent with previous reports on research facilities in many other countries, and not unique to Japanese facilities. A previous study found that 82% of investigators reporting MRI-related studies had experienced IFs during their studies; only 53% indicated that there was a standardized facility procedure to address such discoveries [9]. In a survey of investigators from a leading Canadian neuroimaging center, the investigators emphasized that there were no procedures or guidelines in place to address IFs [21].

In the present study, more than 90% of PIs indicated that option 3 or higher was desirable if sufficient resources were available, suggesting that the majority of PIs considered it appropriate to provide explanations of IFs to participants in advance and to notify them when such findings are discovered. Moreover, the gap between the actual and ideal approaches to IFs implied that PIs were willing to improve the handling of IFs, as long as sufficient resources are provided. When the government funds these facilities, they should subsidize the expenses required to establish policies and guidelines regarding IFs and to recruit specialists for image evaluation to improve IF-related efforts.

### Preferences regarding specialist involvement

Views of investigators and the general population regarding specialist involvement differed considerably. The proportion of investigators who indicated that specialist involvement was desirable (option 4 or higher) was 37.7% in the medical school scenario and 13.3% in the humanities and social sciences department scenario. In contrast, 76.1% and 61.0% of the general population indicated that specialist involvement was desirable in the medical school and humanities and social sciences department scenarios, respectively (option 4 or higher). This discrepancy likely reflects the different views of PIs and the general population regarding the current state of research facilities. The PIs who responded to the questionnaire were already aware that specialists are not always involved in neuroimaging research on humans (Figure 1). In contrast, 80% of the general population expected specialists to check the images in research facilities (Table 3).

Illes and Chin stated that participants may suffer harm if no clinical evaluation is performed when there is a finding that urgently requires further examination [2]. Therefore, they concluded that it would be appropriate to entrust the image readings to a trained specialist [2]. Mamourian also supports this conclusion based on his personal experience after finding a cerebral aneurysm on his images during volunteer work in an MRI study [25]. Based on our findings, we can speculate that the general population expects specialist involvement as a means to avoid false-negative errors. However, Royal and Peterson believe that specialists are more likely to recommend more detailed examinations because they fear liability, causing mental, economical, and temporal burdens on participants with false-positive errors [10]. One potential reason for the difference in opinions reported across the literature is that there are insufficient data to determine how the involvement of a specialist would impact the risk of false-negative and false-positive errors or the participants’ welfare. A challenge for future studies will be to empirically determine detection rates of IFs for specialists and PIs and compare them with subsequently confirmed disease incidence rates.

### Reactive responses vs. proactive responses

Almost all investigators who indicated that specialist involvement was appropriate (option 4 or higher) considered a “reactive” response adequate, only when there was a suspicious finding (option 4). On the other hand, more than half of the general population considered a “proactive” [26] response appropriate, which involves a specialist routinely checking images and actively searching for IFs (option 5 or higher). Furthermore, half of the general population supported a “very proactive” [26] response, i.e., taking images with a clinical device that are not intended for research (option 6). In contrast, none of the investigators chose this option.

At first glance, “proactive” and “very proactive” response models seem superior to a “reactive” response model in terms of the ability to reduce disease oversight (i.e., risk of false-negative errors) by increasing the detection rate of abnormal findings. This approach is taken by a number of research facilities, including the NIH [3, 25, 27–29]. In addition, if a specialist joins a research team in advance and immediately checks all images, s/he could address any situations that require immediate or emergency follow-up [30], which, in turn, will benefit the participant.

However, it is an act that goes beyond formal research objectives to provide a benefit to participants. Therefore, a number of experts question the appropriateness of attempting to reduce the risk of false-negative errors [11, 12]. In their view, the search for health problems that would ordinarily be overlooked deviates from research objectives and is closer to medical practice [11, 12, 31]. Even if such practices are regarded as responsibilities of a physician within a clinical setting, it is difficult to regard them as fundamental responsibilities of the PI [12]. Some believe that results counterbalancing the detection rate for clinically important IFs, believed to be 2% to 8% [14], are unrealistic to expect, and attempts to implement these models are impractical in light of the enormous effort and expenditure required [3, 14, 30, 32, 33]. Our results, which indicate that few investigators support the “proactive” and “very proactive” models, are consistent with the aforementioned views toward PIs’ responsibilities and model feasibility.

We found that the general population tends to support measures that reflect expectations that exceed those of investigators (e.g., the introduction of therapeutic elements into research), suggesting the presence of a “therapeutic misconception” among the general population regarding research. In other words, the general public may erroneously expect therapeutic benefits from participating in a research study [34, 35]. Consent to participate in a study based on misunderstandings such as expectation of benefits or underestimation of risk does not represent true informed consent [1, 7, 11, 12, 26, 36]. As previously demonstrated by a number of neuroimaging studies [4, 22, 23], the present study findings also indicate the tendency of the general public to expect practices similar to medical examinations; in this regard, no distinction is made between research and medical practice. A more detailed interview study targeting the general public and research participants will be required to confirm this finding.

### PI responsibilities under various conditions

Both investigators and the general population supported a more rigorous response in the medical school scenario than in the humanities and social sciences department scenario. Approximately 40% of investigators indicated that specialist involvement would be appropriate (option 4 or higher) in the medical school scenario, as opposed to approximately 13% in the humanities and social sciences department scenario. In contrast, responses that included “no explanation of IFs” (option 1) and “no notification even if IFs were found” (option 2) increased 9-fold in the humanities and social sciences department scenario from 2.9% in the medical school scenario. These findings were unrelated to investigator background. Interestingly, a similar trend was observed in the general population.

Our results are consistent with the conclusions reported by Illes and Chin, and with the recommendations provided by the Detection and Disclosure of Incidental Findings in Neuroimaging Research workshop, which states that there is no single correct approach to IFs, and more than one morally acceptable option may exist depending on the research environment or specialization of the PI [2, 14]. Milstein asserts that PIs may be legally required to provide measures that meet the highest standards of medical practice, regardless of differences in their research environments or specializations compared to those of medical doctors [13]. However, both investigators and the general population in our study demanded more responsibility from PIs with physician licenses at medical institutions, although they were more lenient toward non-licensed PIs at non-medical institutions. As the present study did not provide any insight regarding the reason behind this outcome, further research is warranted.

### Recommendations for handling IFs according to the SRPBS

Based on the relevant publications [7, 31] and our preliminary results, we submitted a recommendation [37] to the SRPBS suggesting the following approach for addressing IFs.

*With respect to all brain images taken during a neuroscience research study conducted by the SRPBS, it is desirable that a licensed physician performs a screening test to appropriately examine if a clear abnormality exists. For the time being, we will treat every abnormality this way on a trial basis to help shed light on unforeseen issues. Re-examination of this method after a year is desirable* [37] *.*

Adopting option 1 or 2, in which no action is taken in response to a finding, essentially denies the PI’s responsibility to the participant. Even though a PI will not be held liable as a physician would, such behavior may be considered irresponsible, as the project is government funded. There is a general consensus among the PIs in our study (Figure 1) and the overseas research community regarding PI responsibility in addressing IFs [6, 7].

In option 3, in which a specialist is not involved, errors associated with false negatives and false positives are expected to increase [31, 37]. Furthermore, the general population in our study considered specialist involvement appropriate regardless of the PI’s specialization or research environment (Figures 2 and 3, and Table 3). The option without specialist involvement is unsuitable for research institutions receiving public funds, given that the majority of the general population desired a more aggressive response in view of the potential harm that can result from false-negative and false-positive errors.

If reducing errors (i.e., false negatives and false positives) is our goal, then option 4 is also inadequate. Although false-positive errors are likely to decrease if a discovered finding is confirmed by a specialist, false-negative errors would still remain high in this setting, because the study is not performed by a specialist. Therefore, options that potentially reduce both types of errors (option 5 or higher) are more desirable. However, option 6 comes exceedingly close to medical practice, and may not only exacerbate “therapeutic misconception” by participants but also go beyond expected fundamental responsibilities of PIs. Although a small proportion of the general population selected option 6 (Figures 2 and 3) in our study, an option that exacerbates “therapeutic misconception” could distort risk-benefit calculations by research participants, making true informed consent impossible [1, 7, 11, 12, 26, 36].

For all brain images taken during the course of research studies conducted as part of the SRPBS, we conclude that image evaluation by a specialist is desirable (option 5). We also recommend that investigators be thorough in obtaining informed consent in an effort to discourage “therapeutic misconception” and that the SRPBS provide economic assistance to research facilities that may have difficulty implementing this option. These recommendations were adopted by the SRPBS and implemented on a trial basis in April 2012. However, consistent with the investigators’ views in the present study (Figures 2 and 3), several studies do not support option 5 [3, 11, 12, 14, 30, 32, 33]. For this reason, future studies on investigator and participant attitudes and IF detection rates are required to evaluate the success of the implemented proposal. Investigation of the impact of IF-addressing measures based on the present proposal, as well as IF detection rates among all images in the SRPBS, is currently underway.

## Conclusions

It is important to note that our study had some sampling limitations. First, study participants included a small number of PIs and investigators. Given that many of them belonged to leading Japanese research facilities with relatively abundant human and economic resources, other options may have been selected, if PIs and investigators from outside the SRPBS were included. Moreover, not all investigators conducted human research using MRI or fMRI. However, our statistical analyses revealed that the type of research or use of imaging equipment did not alter investigator attitudes toward IFs handling procedures.

Second, the general population consisted of individuals who volunteer to participate in studies conducted by a research firm, and who decided to complete our questionnaire. Therefore, we cannot rule out the possibility that the majority of these participants had an interest in neuroimaging research and that they did not truly represent the general public regarding views on IF issues. However, given the likelihood that our participants responded to the questionnaire without fully understanding what an IF is, it is possible that their views are similar to those held by potentially eligible research participants.

The questionnaires used in this study did not address issues of how or who should notify participants in the case of a suspicious IF, despite the relative importance of these issues. This could be considered another limitation. We are currently investigating disclosure conditions at each facility conducting an SRPBS project, as well as the impact of disclosing findings on research participants.

We conducted two questionnaire surveys to clarify i) how IFs are addressed at neuroimaging research facilities in Japan and ii) the views of investigators and the general population regarding the handling of IFs. We found that almost half of the PIs at facilities conducting human research using MRI or fMRI had no or little knowledge regarding the type of approach being employed to handle IFs. Nevertheless, their responses indicated a willingness to improve the current status, as long as sufficient resources are provided. The general population tended to support measures for handling IFs that go beyond those supported by investigators. Both investigators and the general population demanded more responsibility from PIs with physician licenses at medical institutions, although they were more lenient toward non-licensed PIs at non-medical institutions.

This study is empirical in its approach and is not intended to demonstrate clear norms. However, a large portion of the preliminary results obtained from this study was reflected in a proposal regarding approaches to IFs, which was subsequently included in the guidelines for national research projects. In this regard, the present study is of practical significance. We hope that the results of this study will be used as a reference, not only to help establish policies and guidelines in Japan and other countries but also to contribute to the development of neuroimaging research.

## Authors’ information

MF (MS, MPH, PhD) is an Associate Professor of Uehiro Research Division for iPS Cell Ethics at the Center for iPS Cell Research and Application (CiRA), Kyoto University. She has been working as a clinical psychologist and researcher and conducting empirical studies of various bioethical issues such as transplantation, regenerative medicine, and neuroscience. YH (PhD) is an Associate Professor of Philosophy at Ritsumeikan University, Kyoto, Japan. He received a PhD in philosophy from Kyoto University, Kyoto, Japan. His research interests include ethical theories, theories of justice, neuroethics, and sport ethics. ST (PhD) is an Assistant Professor of Office for Promoting Medical Research at Showa University. He holds a PhD in Sociology from Tohoku University. KT (MPH) is a doctoral candidate and project researcher at UT-CBEL (University of Tokyo Center of Biomedical Ethics and Law). She received her MPH from the University of Tokyo. EN (PhD) studied at the Department of History and Philosophy of Science, University of Tokyo. He is presently an Assistant Professor at the Department of Biomedical Ethics at the University of Tokyo Graduate School of Medicine. His area is philosophy of memory, philosophy of science, and neuroethics. AA (MD, PhD) is Professor in the Department of Biomedical Ethics at the University of Tokyo Graduate School of Medicine and Director of UT-CBEL (University of Tokyo Center of Biomedical Ethics and Law). He also serves as Chair of the Ethics Committee at the University of Tokyo, Faculty of Medicine. His research interests include cross-cultural bioethics, informed consent, end-of-life issues, research ethics, bioethics policy, and clinical ethics.

## Abbreviations

IF: Incidental finding
fMRI: Functional magnetic resonance imaging
MEXT: Ministry of Education, Culture, Sports, Science, and Technology
MRI: Magnetic resonance imaging
NIH: National Institutes of Health
PI: Principal investigator
SRPBS: Strategic Research Program for Brain Sciences.
